# The Evasion Mechanisms of Cancer Immunity and Drug Intervention in the Tumor Microenvironment

**DOI:** 10.3389/fphar.2022.868695

**Published:** 2022-05-24

**Authors:** Seong Keun Kim, Sun Wook Cho

**Affiliations:** ^1^ Cellus Inc., Seoul, South Korea; ^2^ Department of Internal Medicine, Seoul National University Hospital, Seoul, South Korea

**Keywords:** tumor microenvironment, immune evasion, tumor associated macrophage, cancer immunity, drug intervention

## Abstract

Recently, in the field of cancer treatment, the paradigm has changed to immunotherapy that activates the immune system to induce cancer attacks. Among them, immune checkpoint inhibitors (ICI) are attracting attention as excellent and continuous clinical results. However, it shows not only limitations such as efficacy only in some patients or some indications, but also side-effects and resistance occur. Therefore, it is necessary to understand the factors of the tumor microenvironment (TME) that affect the efficacy of immunotherapy, that is, the mechanism by which cancer grows while evading or suppressing attacks from the immune system within the TME. Tumors can evade attacks from the immune system through various mechanisms such as restricting antigen recognition, inhibiting the immune system, and inducing T cell exhaustion. In addition, tumors inhibit or evade the immune system by accumulating specific metabolites and signal factors within the TME or limiting the nutrients available to immune cells. In order to overcome the limitations of immunotherapy and develop effective cancer treatments and therapeutic strategies, an approach is needed to understand the functions of cancer and immune cells in an integrated manner based on the TME. In this review, we will examine the effects of the TME on cancer cells and immune cells, especially how cancer cells evade the immune system, and examine anti-cancer strategies based on TME.

## Cancer Immunity and Tumor Immunosurveillance

### The Immune System in Cancer

The immune system maintains its defense against infected antigens and self-antigens through an appropriate balance between activation and inhibition of immune response. At its core, this process involves receptor-ligand binding between T cells activated by antigens and various cells within the immune system, and the immune response is known to play an important role in carcinogenesis and anti-tumor immunity (Naidoo et al., 2014).

The immune response is the intraorganism defense mechanism that responds to threats to self. This defensive reaction takes place in three stages: recognition, processing, and reaction. Non-self elements are recognized as foreign substances by cells or active molecules involved in the immune response. Cells or active molecules involved in innate immunity recognize molecular patterns of non-self antigens. Innate immunity includes cells such as natural killer (NK) cells, macrophages, dendritic cells (DC), and neutrophils, as well as active molecules such as complement system in serum. The lymphocytes and antibodies involved in adaptive immunity have the ability to recognize the structure of non-self antigens or amino acid sequences in detail with high accuracy. In order to induce an effective immune response that can fight infectious diseases or cancer, cells or active molecules in the innate immune system and the adaptive immune system must interact in a coordinated manner. When cells from both systems respond properly at the early stage of cancer, cancer cells can be removed and the tissue returns to its normal state (Pulendran et al., 1997; Medzhitov and Janeway, 2002).

However, cancer cells grow and spontaneously proliferate faster than the immune system can handle through strategies that deceive the immune system and prevent attacks from immune cells. To do this, cancer cells begin to build a microenvironment starting at the time of cancer occurrence, and in most cases, when a cancer mass is found, a tumor microenvironment (TME) is present, making it difficult for the immune system to efficiently eliminate cancer cells (Labani-Motlagh et al., 2020).

### Tumor Immunosurveillance

Cancer immunology first began in 1957 when Burnet proposed the concept of tumor immunosurveillance (Burnet, 1957). Tumor immunosurveillance is when tumor cells are first recognized by T cells at the occurrence and growth stage, and then are killed by immune cells and secreted interferon-gamma (IFN-γ). In addition, tumor cells actively induce T-cell tolerance, resulting in an immunoediting process that facilitates tumor re-distribution and growth (Dunn et al., 2002). Accordingly, in cancer immunology, efforts have been made to develop immunotherapy against tumors by targeting the interactions between cancer cells and immune cells. Many recent studies have revealed that various cancers diminish normal immunity from having effective anti-cancer activity, as exemplified by findings of decreased absolute counts of lymphocytes (Kuss et al., 2005), an increasd regulatory T cells (Schaefer et al., 2005; Strauss et al., 2007) and tumor-associated macrophages (Li et al., 2002), down-regulation of antigen expression in tumor-associated cells (Grandis et al., 2000), and apoptosis of cytotoxic T cells (Whiteside, 2005). In addition, direct inhibition of the immune mechanism occurs due to vascular endothelial growth factor (Gabrilovich et al., 1996), prostaglandin E2 (Benefield et al., 1996; Schroeder et al., 2004), transforming growth factor-β (TGF- β) (Qin et al., 2001), and interleukin (IL)-10 secreted from cancer cells (Dennis et al., 2013), further decreasing the function of anti-cancer immunity. The immune response is also indirectly involved in tumor development through a reduction of the Th1 response, which induces anti-cancer effects, cytotoxic T cell differentiation, and an increase in the antagonistic Th2 response (Agada et al., 2009).

Interestingly, direct and indirect studies have reported that chemotherapy and radiation therapy, which are direct treatments for cancer and its progression, also regulate the immune system’s response against cancer cells. When external radiation therapy is performed for malignant melanoma, lymphoma, and renal cancer, improvement also occurs outside the treated area, and a recent report showed that these effects of cancer treatment might be caused by the activation of cancer-specific antibodies and T cells (Postow et al., 2012; Stamell et al., 2013). It has also been reported that the immune response is suppressed in high-dose chemotherapy, while in low-dose chemotherapy, the anti-cancer immune response is stimulated or induced (Zitvogel et al., 2008; Galluzzi et al., 2015).

Cancer immunotherapy is research on the effects of existing cytotoxic chemotherapy and radiation therapy on the host immune system based on an understanding of the relationship between cancer and the immune system. Although cancer immunotherapy has been attempted since 1990 in preclinical and early clinical studies with immunostimulating cytokine therapy, cancer vaccine therapy, and adoptive T cell therapy, it has not been introduced into practical use in clinical settings due to the local delivery of drug, systemic toxicity, and a lack of remarkable treatment results. Nevertheless, there has recently been a rapid increase in interest in immunotherapy, as exemplified by research on the involvement of immune mechanisms in the treatment of cetuximab (Holubec et al., 2016), diverse research results on immune evasion mechanisms, and early clinical studies of monoclonal antibodies to block immune checkpoints that showed satisfactory results not only for malignant melanoma, but also for head and neck cancer and non-small cell lung cancer, which is a non-immune solid cancer (Bauman and Ferris, 2014).

## Tumor Microenvironment and Immunotherapy

### Cancer and Immune Cells

Tumor tissue, besides tumor cell also includes a variety of other cell types such as T cells, NK cells, macrophages, fibroblasts, dendritic cells, and adipocytes (Andrejeva and Rathmell, 2017). The tumor microenvironment is characterized by cancer cells that exhibit diverse metabolic variations due to their heterogeneity, in accordance with their proliferation and metastasis. Therefore, improvement of tumor immunotherapy is tightly linked with improvement of our understanding of the TME.

Cancer cells change through mutations or abnormal regulation, while immune cells change through specific mechanisms related to external invasion. In addition, cancer cells require sufficient nutrient uptake to maintain their metabolism and regulate their growth, whereas immune cells are relatively flexible compared to cancer cells because they can maintain their function through a balance of various metabolic systems. Thus, studies aiming to inhibit cancer metabolism by regulating the signaling pathway of immune cells through interactions between immune cells and cancer cells have recently been highlighted. Cancer cells affect the metabolism of T cells through the activation and proliferation of immune cells, depletion of glucose (Kleffel et al., 2015; Shi et al., 2019) and amino acids (e.g., leucine (Sinclair et al., 2013; Patel et al., 2019), serin (Munn et al., 1998; Munn et al., 1999; Srivastava et al., 2010), tryptophan (Munn et al., 1999; Munn et al., 2005), and glutamine (Cheng et al., 2011; Wang et al., 2011; Leone et al., 2019)), conditions of high acidity and high lactic acid levels, and up-regulation of immune checkpoints.

Cancer cells affect the metabolism of T cells in various ways, and through the activation and proliferation of immune cells, the depletion of glucose and amino acids, high acidity and lactate, and upregulated immune checkpoint, consequently inhibiting glycolysis, thereby affecting the metabolism of T cells. To summarize the metabolic mechanism between cancer cells and immune cells, M1 macrophages, effector T cells, and normal cancer cells regulate proliferation and anabolism using a high proportion of glycolysis and glutaminolysis to synthesize proteins, nucleic acids, and amino acids, while M2 macrophages, regulatory T cells, and quiescent cancer cells regulate catabolism using the oxidation of fatty acids to synthesize ATP (Andrejeva and Rathmell, 2017; Kouidhi et al., 2018). Among them, tumor-associated macrophages, corresponding to M2 macrophages, are the most abundant immune cells in the TME (accounting for 50%), and mainly affect cancer progression and resistance by supplying or supporting nutrients to malignant cancer cells. In particular, because tumor-associated macrophages are responsive to pharmacological agents that improve bacterial and oxidative function (Hossain et al., 2015; Jayaprakash et al., 2018; Duan and Luo, 2021), mechanisms of regulating their metabolism are highly promising targets for novel cancer treatment. Therefore, recent studies have considered the mechanisms through which tumor-associated macrophages are involved in the overall metabolism of the TME for this goal (Vitale et al., 2019).

### Cancer and Immunotherapy

In recent years, substantial advances have been made in various immune-based treatments, including the administration of specific cytokines, antibodies to immune checkpoints, and cell therapies such as CAR-T and CAR-NK, as a new paradigm of cancer treatment. These advances can be mainly summarized as attempts to modulate immune cell activity through the regulation of T cells using adoptive cell delivery and monoclonal antibodies (Hosseinkhani et al., 2020; Marofi et al., 2021). To date, among these immune-based treatments, immune checkpoint inhibitors are most widely used as an effective immunotherapy for a variety of solid tumors and malignant hematologic tumors. Immune checkpoint inhibitors are defined as drugs that upregulate the immune response using monoclonal antibodies targeting CTLA4 and PD-1, which are located on the cellular membrane of T cells and cancer cells (Waldman et al., 2020). Although there have been many studies on the mechanism and utilization of immunotherapy with immune checkpoint inhibitors, immunotherapy targeting only these single antigens has limitations due to the high immunosuppression of the TME and the low immunogenicity of cancer cells. A recent study showed that a combination of anti-PD1 and anti-CTLA4 to treat metastatic melanoma and non-small cell lung carcinoma (NSCLC) resulted in a better response than was achieved with conventional single antibody immunotherapy, as shown by increased patient life expectancy and the inhibition of new metastasis (Antonia et al., 2016; Hellmann et al., 2017). In addition, combination of immune checkpoint inhibitors with other chemotherapy agents, has recently attracted attention as a new strategy for anti-cancer treatment (Lee et al., 2020a). Nevertheless, tumor-infiltrating lymphocytes (TILs) need to overcome not only the immune checkpoints, but also a wide range of metabolic checkpoints within the TME that weaken their functions (Kouidhi et al., 2018). Thus, it is important to improve our comprehensive understanding of the mechanisms that reduce anti-cancer immunity within the metabolically hostile TME.

In fact, cancer cells can regulate immune cells by upregulating nutrient uptake and metabolite production, and as a result, constructing an immunosuppressive TME that promotes cancer cell growth and immune evasion. However, some recent studies have suggested that combining the immune checkpoint inhibitors and metabolic regulating agents can decrease cancer cell metabolism more effectively than either approach used in isolation (Murciano-Goroff et al., 2020; Weng et al., 2021). Caution is needed to ensure that treatments of this type only target specific tumor sites to prevent side effects such as systemic toxicity. Although cancer metabolism has been studied long enough to be considered the basis of cancer, active research is still undergoing and the mechanisms of cancer metabolism remain to be fully elucidated. Therefore, various aspects of cancer metabolism and its metabolites are noteworthy for future cancer research (Stine et al., 2022).

## Cancer Immune Cycle and Immune Evasion

### Cancer-Immune Cycle

**FIGURE 1:**
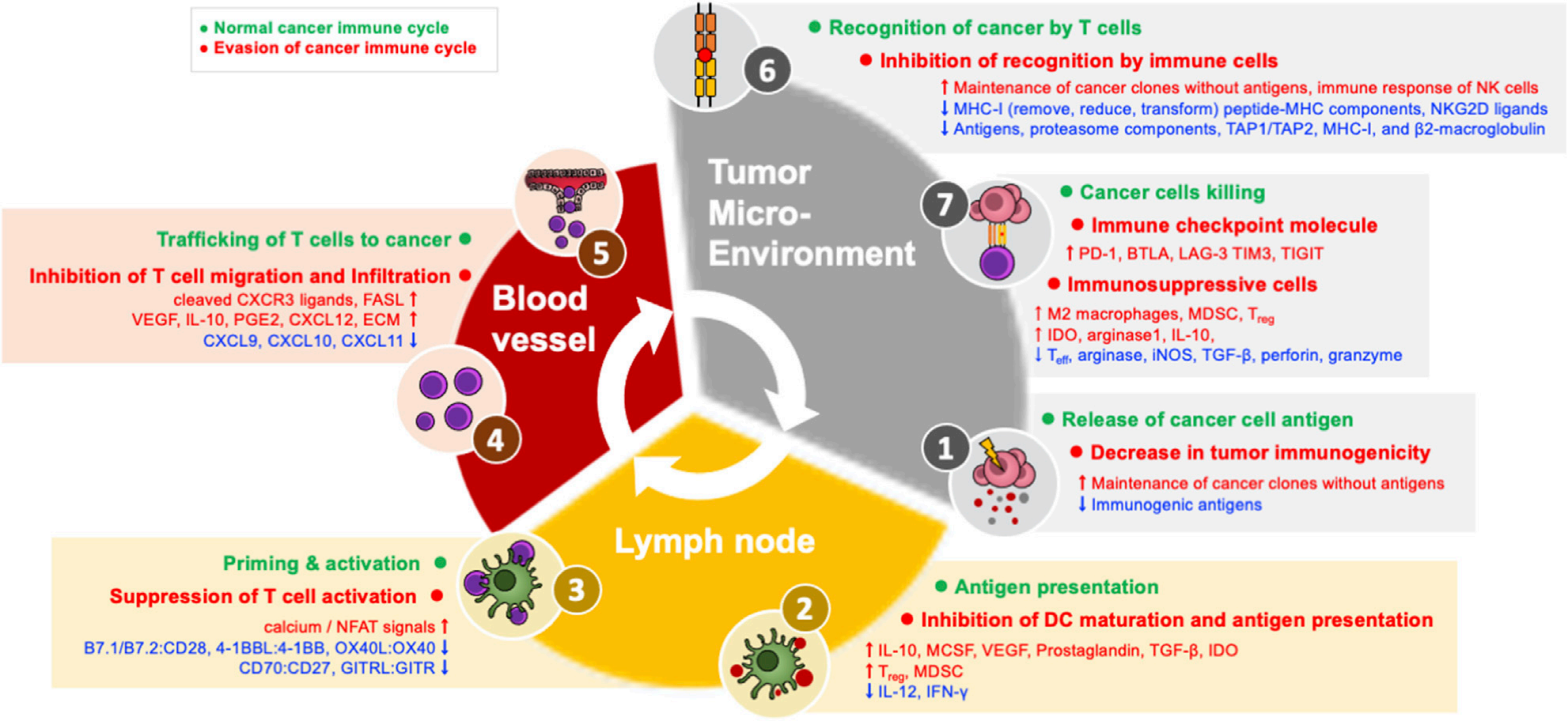
Evasion machanisms in the cancer immune cycle.

In order for an anti-cancer immune response to effectively kill cancer cells, a series of steps must be repeated and amplified. This series of steps is called the cancer-immune cycle: 1) Neoantigens produced during tumor formation are released from dead cancer cells, and dendritic cells (DCs) acquire them and move to the nearest draining lymph node (DLN). 2) DCs present the acquired antigen to T cells through major histocompatibility class I (MHC-I) and MHC-II molecules. 3) Effector T cells are activated by recognizing this antigen (Chen and Mellman, 2013; Motz and Coukos, 2013; Kim and Chen, 2016; Chen and Mellman, 2017; Patel and Minn, 2018). 4) Antigen-recognizing cancer-specific T cells in the DLN express the cell adhesion molecules and chemokine receptors necessary for migration and infiltration into the tumor, and then leave the DLN and move through the blood toward the tumor tissue. 5) These T cells then infiltrate into the tumor and 6) recognize and bind the MHC-I–antigen complex presented by cancer cells through the T cell receptor (TCR). 7) Through these processes, cancer cells are finally killed. CD8^+^ T cells kill cancer cells through granule-exocytosis mechanisms mediated by perforin and granzyme and interactions between apoptotic ligands-receptors such as FasL-Fas. When cancer cells die and additional neoantigens are released, the immune reaction continues the cycle again from the first step, and it is amplified compared to the previous reaction; for this reason, this mechanism is termed the cancer-immune cycle (Figure 1) (Chen and Mellman, 2013; Motz and Coukos, 2013; Kim and Chen, 2016; Patel and Minn, 2018). In cancer patients, at least one of these steps is defective and the cancer-immune cycle does not work properly.

### The Evasion Mechanism of the Cancer-Immune Cycle in “Cold” Tumors

Successful antigen recognition and activation (steps 1–3) of T cells, which are the early stages of the cancer-immune cycle, and infiltration (steps 4–5) of T cells into the tumor are essential conditions for “hot” tumors. This type of immune infiltration is usually characterized by reactivity to immune checkpoint inhibitors. In contrast, in “cold” tumors, characterized by immune non-infiltration, there is a defect in the initial stage of the cancer-immune cycle, and most cold tumors do not respond to immune checkpoint inhibitors. Therefore, antigen recognition and migration of T cells to the tumor can be assumed to be the main factors influencing reactivity to immune checkpoint inhibitors.

(1) Decrease in tumor immunogenicity (evasion of step 1): During tumor formation, immunosurveillance removes cancer clones that express strong immunogenic neoantigens. At this point, the tumor evades anti-cancer immune responses by eliminating immunogenic antigens or maintaining cancer clones without cancer antigens so that they are not recognized by T cells. In other words, cancer clones that evade immunosurveillance have fewer immunogenic antigens (Dunn et al., 2004; Coulie et al., 2014; Kim and Chen, 2016).
(2) Inhibition of dendritic cell maturation (evasion of step 2): Damage-associated molecular patterns, such as ATP and high-mobility group box 1 (HMGB1) (Zhu et al., 2009), released from dead cancer cells can induce DC maturation. Cancer inhibits DC maturation through tumor derived-factors such as IL-10 (Williams et al., 2004), macrophage colony-stimulating factor (MCSF) (Nefedova et al., 2004), vascular endothelial growth factor (VEGF) (Gabrilovich et al., 1996), prostaglandin (Sá-Nunes et al., 2007), TGF-β (Zong et al., 2016), and indoleamine 2,3-dioxygenase (IDO) (Munn and Mellor, 2016). In addition, immunosuppressive cells in the TME, such as T_reg_ and myeloid-derived suppressor cells (MDSCs) express inhibitory factors that suppress DC maturation, reducing the expression of MHC and co-stimulatory factors in DCs, resulting in reduced production of inflammatory cytokines, such as IL-12, and ultimately inhibiting the proliferation of T cells and IFN-γ (Dunn et al., 2004; Hwang et al., 2005; Novitskiy et al., 2008; Steinman, 2012; Lindau et al., 2013; Kim and Chen, 2016; Li et al., 2020).
(3) Suppression of T cell activity (evasion of step 3): For full activation of T cells, both antigen recognition and co-stimulatory signals are required. Co-stimulatory interactions between DC and T cells include B7.1/B7.2:CD28, 4-1BBL:4-1BB, OX40L:OX40, CD70:CD27, and GITRL:GITR. These co-stimulatory interactions promote proliferation, differentiation, survival, cytotoxic function, memory formation, and cytokine generation of T cells. Tumors inhibit the activity of T cells by reducing the expression of co-stimulatory factors and MHC, limiting the co-stimulation required for T cells. When the TCR is activated without co-stimulation, excessive activity of calcium/nuclear factor of activated T-cell (NFAT) signals induces the expression of negative modulating factors and T cells become unresponsive (T cell anergy) (Gimmi et al., 1993; Macián et al., 2002; Williams et al., 2006; Chen and Flies, 2013).
(4) Inhibition of T cell migration and infiltration (evasion of steps 4-5): T cells express chemokine receptors such as CXCR3 on the cell surface in response to IFN-γ during the activation process (Kuo et al., 2018). As a major evasion mechanism at this stage, cancer cells reduce the expression of CXCR3 ligands such as CXCL9, CXCL10, and CXCL11, and/or carry out posttranslational modification or decomposition of CXCR3 ligands, thereby inhibiting the migration of CD8^+^ T cells to the tumor (Karin, 2020). These fragments of cleaved CXCR3 ligands may also act as antagonists of the receptor. As another mechanism for inhibiting the migration of T cells, tumors transform nearby blood vessels. Tumors produce neoplastic factors such as VEGF, reducing the expression of adherent factors in endothelial cells (ECs), which are important for the migration of T cells (Gupta and Qin, 2003; Zarychta and Ruszkowska-Ciastek, 2022). In addition, IL-10 and prostaglandin E2, which are immunosuppressive factors, are produced and promote Fas ligand expression along with VEGF, thereby inducing apoptosis of CD8^+^ T cells infiltrating the tumor (Motz et al., 2014; Lee et al., 2020b). Moreover, the endothelin-B receptor expression of tumor ECs is increased to inhibit the migration of T cells (Motz et al., 2014; Slaney et al., 2014; Joyce and Fearon, 2015; Mikucki et al., 2015). However, even if CD8^+^ T cells move toward tumor tissue, they may not be able to infiltrate the center of the tumor. This is because immunosuppressive immune cells and cancer-associated fibroblast (CAF) around the tumor produce extracellular matrix (ECM) proteins to physically suppress T cells or produce chemokines such as CXCL12, inhibiting the migration of T cells to tumors. In fact, analyses of human lung cancer tissue have confirmed that fibroblasts or collagen accumulates in the tumor substrate to prevent interactions between T cells and tumor cells (Salmon et al., 2012; Turley et al., 2015).

### The Evasion Mechanism of the Cancer-Immune Cycle in “Hot” Tumors

When the initial 5 stages of the cancer-immune cycle are successfully performed, immune infiltration into the TME is formed. Similar to the initial stage of the cancer-immune cycle, the mechanism of immune evasion of the last stage also takes place in the TME. PD-L1:PD-1 interactions are only one of several causes of immunosuppression, so reactivity to immune checkpoint inhibitors is sometimes absent even when the TME has immune infiltration. In addition, even in patients who initially respond to an immune checkpoint inhibitor, secondary immune evasion can occur when the tumor inhibits immune reaction in response to attacks by T cells.

(1) Inhibition of recognition by immune cells (evasion of step 6): Cancer cells remove, reduce, or transform MHC-I on the surface of cancer cells as a mechanism to evade recognition by T cells. Cancer cells directly regulate MHC-I genes or proteins or indirectly inhibit peptide-MHC components (Dhatchinamoorthy et al., 2021). In addition, cancer cells downregulate the expression of antigens, proteasome components, TAP1/TAP2, MHC-I, and β2-microglobulin through mutation, genetic loss, transcription inhibition, or epigenetic inhibition of gene expression (Taylor and Balko, 2022). Recent cancer genome studies have confirmed that the reduction of peptide-MHC-I expression on the surface of cancer cells due to somatic mutations in human leukocyte antigen (Campoli and Ferrone, 2008; Lawrence et al., 2013). Even if cancer cells can evade recognition by T cells through reduced MHC-I expression, NK cells cannot be evaded. This is because NK cells can induce an immune response to abnormal cells by recognizing the degree of MHC-I expression on the cell surface. As an alternative to this, cancer cells release ligands to NKG2D, an active receptor of NK cells, to evade lysis by NK cells (Ljunggren and Kärre, 1990; Groh et al., 2002; Terry et al., 2019; Hu et al., 2020).
(2) Immune checkpoint molecule expression (evasion of step 7): The CD8^+^ T cells that infiltrate a tumor can simultaneously express several additional co-inhibitory receptors in addition to PD-1, including B and T lymphocyte attenuator (BTLA), lymphocyte activation gene 3 protein (LAG-3), T-cell immunoglobulin domain, mucin domain-3 (TIM3), T-cell immunoglobulin, and immunoreceptor tyrosine-based inhibitory motif domain (TIGIT). Other co-inhibitory receptors are expressed simultaneously, and T cells become exhausted T cells (T_ex_) that are unresponsive to immune checkpoint inhibitors.
(3) Immunosuppressive cells (evasion of step 7): Immunosuppressive cells in the TME are also an important mechanism of immune evasion. The TME induces macrophages to differentiate into M2-type tumor-associated macrophages that promote tumor formation, and tumor-associated macrophages generate IL-10 instead of IL-12 to inhibit the CD8^+^ T cell response. Tumor-associated macrophages directly inhibit immune checkpoint inhibitor responses by removing anti-PD-1 antibodies from PD-1^+^ CD8^+^ T cells in an FcγR-dependent manner (Garris et al., 2018; Chen et al., 2019). MDSCs are a group of heterogeneous cells that can strongly inhibit the T_eff_ response and induce T_reg_. MDSCs inhibit the immune response by generating arginase, inducible nitric oxide synthase (iNOS), and TGF-β. In particular, TGF-β inhibits the cytotoxic activity of cytotoxic T cells and NK cells by reducing the expression of cytotoxic factors such as perforin and granzyme. T_reg_ cells are among the immunosuppressive cells of the TME; when their number increases, they are known to inhibit the CD8^+^ T cell response and promote tumor progression. A high T_reg_ frequency is generally associated with a poor prognosis. For example, strong anti-cancer immune responses have been reported in T_reg_-deficient mouse models, and these results suggest that T_reg_ cells play an important role in inhibiting anti-cancer immunity (Han et al., 2019; Verma et al., 2019). IDO, an immunosuppressive enzyme expressed in myeloid cells and various cancer cells, induces kynurenine, which is a tryptophan metabolite that exerts immunosuppressive actions. This is known to strongly suppress T_eff_ function while promoting the generation and activity of T_reg_ and MDSCs. Another immunosuppressive enzyme, arginase 1, inhibits the function of DCs through cooperation with the IDO mechanism. Other metabolites (e.g., glucose consumption, lactate generation, cholesterol metabolism) and inflammatory mechanisms (e.g., cyclooxygenase-2/prostaglandin E2) are also known to affect cancer cells and immune cells (Rodriguez et al., 2004; Ohta et al., 2006; Kalinski, 2012; Munn and Mellor, 2013).

## Cancer Metabolism and Immune Evasion

### Metabolic Diversity in Cancer

Cancer transforms its own metabolic processes to maintain rapid growth and survival. Normal cells generate energy through oxidative phosphorylation (OXPHOS) in mitochondria, while cancer cells generate ATP through aerobic glycolysis even in an environment with sufficient oxygen, which is known as the Warburg effect (Warburg et al., 1927). The Warburg effect promotes the growth and survival of cancer cells, facilitates adaptation to changes in oxygen concentration in tissues, and uses intermediate products of glycolysis to maintain cell proliferation. In addition, lactate produced through glycolysis maintains TME oxidized, contributing to cancer infiltration and immune evasion (de la Cruz-López et al., 2019). However, recent studies have shown that OXPHOS and mitochondrial metabolic processes are also important for cancer metabolism. Some tumors increase OXPHOS by oxidation of glucose, protein, amino acids (e.g., glutamine or tryptophan), and fatty acids, and some tumors use waste products such as ammonia and lactate as energy sources (LeBleu et al., 2014; Yang et al., 2014; Hensley et al., 2016; Qu et al., 2016; Faubert et al., 2017; Spinelli et al., 2017; Badur and au, 2018; Corbet et al., 2018; Kuo and Ann, 2018; Pavlova et al., 2018; Porporato et al., 2018). These changes in cancer metabolism are controlled by carcinogenic mutations (e.g., MYC, phosphatase and tensin homologue; PTEN, AKT serine/threonine kinase, and phosphoinositide 3-kinase; PI3K). In addition, carcinogenic mutations are controlled by changes in surrounding factors or specific metabolic enzymes (e.g., isocitrate dehydrogenase 1; IDH1, succinate dehydrogenase; SDH, and IDO) (Buescher and Driggers, 2016; Maj et al., 2017; Reznik et al., 2018; Wellenstein and de Visser, 2018). Changes in metabolite concentration activate specific metabolic pathways, affect cell function through metabolite-mediated signaling pathways, and change the epigenetics of cancer cells. For this reason, even within a single tumor tissue, metabolic changes vary from cancer cell to cancer cell, and the use of nutrients in the TME is also quite different (Lane et al., 2020). These complex and diverse tumor metabolic changes construct a TME that threatens the survival of immune cells, and in response, immune cells activate specific metabolism to control their own survival and cancer cell growth. Therefore, further research should aim to elucidate the diversity of tumor metabolism in order to develop effective anti-cancer immunotherapy (Hu et al., 2013; Crompton et al., 2015; Hensley et al., 2016; Pavlova and Thompson, 2016; Grzes et al., 2017; Palm et al., 2017; Reid et al., 2017; Badur and au, 2018; Wellenstein and de Visser, 2018).

### TME Metabolism Affecting Anti-Cancer Immunity

The concentration of metabolites such as glucose, lactate, and glutamine in the TME affects the function and activity of tumor-infiltrating immune cells. Cancer cells deplete glucose in the TME, inhibiting the anticancer immunity of T cells, NK cells, macrophages, and DCs that use glucose for anti-cancer activity (Renner et al., 2017; Xia et al., 2021). Mechanistically, glycolysis is necessary to regulate IFNγ production by T cells, and the restricted use of glucose in the TME limits Ca^2+^ signaling, glycolysis, and cytokine production of TILs (Rangel Rivera et al., 2021; Zhang et al., 2021). In fact, resistance to T cell therapy appears in cancer patients with overexpressed glycolysis enzymes. If the available glucose concentration is increased by inhibiting glycolysis in the tumor, T_eff_ cells are activated, and the production of immunosuppressive cytokines by cancer cells is inhibited, thereby improving anticancer immunity (Maj et al., 2017; Le Bourgeois et al., 2018; Leone and Powell, 2021; Traba et al., 2021; Watson et al., 2021; Kumagai et al., 2022). Like glucose, lactate produced by the glycolysis of cancer cells or immune cells also inhibits immune cell function. Lactate inhibits the function of T_eff_ cells, but not T_reg_ cells, ultimately inducing an immunosuppressive environment. Glutamine in the TME also allows T_reg_ cells to accumulate, rather than the Th1 response, if its concentration is limited. Reducing glucose and glutamine in the TME limits UDP-GlcNAc synthesis by T cells and promotes differentiation into T_reg_ rather than Th17 (Chang et al., 2015; Angelin et al., 2017; Le Bourgeois et al., 2018; Watson et al., 2021; Xia et al., 2021; Kumagai et al., 2022; Watson and Delgoffe, 2022). Since cytotoxic cells such as CD8^+^ T cells and NK cells are sensitive to amino acid restrictions, their function is suppressed when glutamine, serine, and glycine are depleted or when branched-chain amino acids, especially leucine and isoleucine, are restricted (Gentles et al., 2015; Badur and au, 2018). Therefore, restrictions on the use of nutrients in immune cells are immunologically associated with cancers that do not undergo immune infiltration (cold tumors) or malignant tumors.

Tumor-infiltrated immunosuppressive cells (T_reg_ cells, tolerogenic DCs, and MDSCs) and cancer vascular endothelial cells also reduce nutrients in the TME, potentially contributing to an immunosuppressive environment. T_reg_ cells act competitively against glucose, inducing replicative senescence of CD4^+^ and CD8^+^ T cells. The activity of TLR8 hinders the corresponding action of T_reg_ cells, thereby improving anticancer immunity. In addition, T_reg_ cells in the TME convert ATP into adenosine, inhibiting the activity of immune cells in tumors (Lindau et al., 2013; Peixoto et al., 2019; Haist et al., 2021).

Taken together, the immune state in the TME is determined by the competitive action against nutrients, the accumulation of metabolites that inhibit immune response, and cellular signaling that changes metabolic processes. These changes in the TME ultimately induce the recruitment of tumor-associated macrophages, which secrete various cytokines that induce angiogenesis, metastasis of cancer cells, and immunosuppression. Understanding this process will be important for the success of anti-cancer immunotherapy.

## Role of EMT in Cancer Immune Evasion

The TME is composed of various cells around cancer cells as mentioned above. These various cells in TME play a complicated role in inducing epithelial-mesenchymal transition (EMT) in tumor progression and metastasis (Gabrilovich and Nagaraj, 2009; Sangaletti et al., 2016). EMT mechanistically was studied in some cancers such as breast (Shan et al., 2015; Yang et al., 2019), thyroid (Lin et al., 2018), and colon cancer (Ma et al., 2019), and related signaling pathways were found PI3K/AKT/PKB, MAPK/ERK (Ma et al., 2019; Yang et al., 2019), WNT/β-catenin (Shan et al., 2015), and NF-κB (Lin et al., 2018) pathways.

In tumor microenvironments, the cells supporting cancer cells, such as CAF, CD4^+^ T cells, Treg, MDSC, and TAM, inhibit the epithelial state of tumor, promote and activate the mesenchymal state. In this case, these cells also inhibit anti-cancer immune cells such as CD8^+^ T cells, NK cells, and activated M1 macrophages. Cancer cells that come up to the mesenchymal state can up-regulate the expression and activation of various immune cells that affect tumor progression. Treg, M2 macrophages, and MDSC activated by cancer cells directly inhibit the anti-cancer function of T cells and NK cells to support tumor progression (Gabrilovich and Nagaraj, 2009; Kerkar and Restifo, 2012; Gajewski et al., 2013). These EMT-mediated communications between cancer cells and immune cells have been the object of intensive investigation in recent years, considering the potential efficacy of immunotherapy for various cancer, suggesting that EMT increases evasion of cancer cell removal processes by immune cells. In fact, melanoma cells expressing SNAIL have been reported to secrete TGF-β and thrombospondin 1 to induce the activity of Treg and reduce the ability of dendritic cells to present antigens (Kudo-Saito et al., 2009). These melanoma cells expressing SNAIL showed resistance to immunotherapy, and inhibition of SNAIL promoted recovery of anti-cancer immune response and sensitivity to immunotherapy. EMT-mediated immune evasion effects have also been observed in other cancer. While a large number of CD8^+^ T cells infiltrated into the epithelial state of breast cancer, there were many Treg cells and TAM in the mesenchymal state of breast cancer (Kudo-Saito et al., 2009). In addition, only epithelial state tumors were inhibited by anti-CTLA4 ICI treatment, while mesenchymal state tumors were resistant to the same treatment. Treg and TAM continued to infiltrate even in mixed tumors with only 10% of mesenchymal state tumors, and anti-CTLA4 ICI showed no effect (Dongre et al., 2017). The resistance of mesenchymal state cancer cells to attack cytotoxic T cells was also demonstrated in human breast cancer cell lines (Akalay et al., 2013). It has been reported that MCF-7 cells activated the response of T cells when co-cultured conditions with T cells, however, T cell function decreases when MCF-7 cells express SNAIL (Akalay et al., 2013). Even though these studies clearly demonstrate the relationship between EMT and immunosurveillance, the mechanism for immune evasion of mesenchymal state tumors has not been understood clearly.

Nevertheless, considering the communication system within the tumor microenvironment so far revealed, EMT induction seems to be mainly controlled by multiple cytokines and chemokines exchanged between tumors and various cells in the tumor microenvironment. EMT occurring cancer cells secrete TGF-β, which stimulates resident immune cells in tumor microenvironments to promote the secretion of multiple cytokines and chemokines to evade immunity. For example, melanoma cells expressing SNAIL secrete chemokine CCL2, which leads to the secretion of LCN2 (lipocalin 2). CCL2 and LCN2 reduce the MHC-I expression of T cells and induce the expression of cancer cell immunosuppressive molecules such as PD-L1 to induce exhaustion of cytotoxic T cells (Kudo-Saito et al., 2013). The expression of PD-L1 in cancer cells induced by EMT consequently evaded attacks on CD8^+^ T cells (Dongre et al., 2017; Noman et al., 2017), increasing cancer metastasis, which was shown to inhibit immune response even in EMT occurred NSCLC patients through increased expression of various immune checkpoint proteins and increased Treg (Celià-Terrassa et al., 2012; Tripathi et al., 2016). Prostate cancer cells produce CXCL1, its receptor CXCR1 downstream signaling produces LCN2 in neutrophils (Lu et al., 2019). The CXCL1-LCN2 axis activates Src signaling and leads to EMT and contributes to tumor progression (Lu et al., 2019). Furthermore, in pancreatic cancer CXCL8 causes cell invasion and promotes metastases (Wang et al., 2017). Similarly in gastric cancer, neutrophils like cells expressed CXCL8 and induced EMT through CXCR1/CXCR2 receptors (Yang et al., 2016; Bouris et al., 2018). Another chemokine related to tumor progression and metastasis, CXCL16 has been reported that could promote brain metastases in breast cancer (Chung et al., 2017). In addition, it has been reported that EMT causes liver metastasis in colorectal cancer patients (Matsushita et al., 2012). High expression of CXCL16 was associated with M2 macrophage- and angiogenesis-related genes which were poor prognostic factors including a higher TNM staging and the BRAFV600E mutation (Kim et al., 2019). Several CC chemokines CCL5 and CCL18 also promote EMT, cell migration and invasion in co-culture experiments involving TAMs and different cancer cells (Moody et al., 2005; Shioiri et al., 2006). Furthermore, CCL20 of monocyte-derived macrophages induces EMT and induces tumor metastases in hepatoma cells (Yang et al., 2016; Saijo et al., 2018). In addition, fibroblasts induced CCL17/CCL22 plays a critical role in the malignant progression of prostate, breast and hepatocellular cancer (Cheng et al., 2017).

It rarely happens that cancer cells completely transition to a mesenchymal state during human carcinogenesis. Although many studies are underway on the role of tumor microenvironments in inducing EMT, the mechanisms for occurring partial EMT are still unclear. Understanding the signaling pathway for maintaining this condition might be important to develop more effective anti-cancer strategies for most cancers.

## Anti-Cancer Therapeutic Strategy Based on TME

Recently, research on immunotherapy that treats cancer by controlling the immune system is being actively conducted. With the development of immune checkpoint inhibitors at the forefront, the results of various clinical approaches using this immunotherapy have been reported. In particular, research continues to further increase the survival period of cancer patients and maintain the continuity of treatment through combination therapy of various anti-cancer drugs with immune checkpoint inhibitors. However, as mentioned earlier, TME upregulate the expression and secretion of immunosuppressive signal proteins such as IL-10 (Williams et al., 2004), IDO (Munn and Mellor, 2016), and TGF-β (Zong et al., 2016). Through this, normal immune cells for removing cancer cells are transformed to immunosuppressive cells such as T_reg_ (Wang, 2006; Verma et al., 2019) and MDSC (De Cicco et al., 2020; Yang et al., 2020), resulting in down-regulate the anti-cancer effects of drugs.

A new strategy for immunotherapy is to change the cancer immunity based on the TME and treat it. Various studies earlier, it has shown that the TME has an effect on the anticancer effect of immune checkpoint inhibitors (Ciciola et al., 2020; Tang et al., 2021). From this point of view, more recently, various therapeutic approaches have been tried to increase the anti-cancer effect through the combination treatment of TME inhibitors and immune checkpoint inhibitors that suppress mediates in the TME affecting the proliferation of cancer (Table 1).

**TABLE 1:** Combination TME-targeted therapy and Immunotherapy.

Drug	Mechanism	Indication	Clinical phase	Combination with ICI	Clinical trial no.
ADG106	anti-CD137 mAb	Metastatic NSCLC	Ib/II	Nivolumab	NCT05236608
Anlotinib (AL3818)	VEGFR2 inhibitor	NSCLC, SCC, Solid tumor, Soft tissue sarcoma	I/II	Nivolumab	NCT04165330
Apatinib	VEGFR2 inhibitor	Cancer	I	Nivolumab	NCT03396211
Aspirin	COX-1/2 inhibitor	Ovarian cancer	II	Atezolizumab	NCT02659384
		TNBC	I	Avelumab	NCT04188119
		Ovarian cancer	II	Bevacizumab	NCT02659384
		Advanced TIL-negative solid tumors	I	Ipilimumab	NCT03728179
		Advanced TIL-negative solid tumors	I	Nivolumab	NCT03728179
		Head and Neck cancer	I	Pembrolizumab	NCT03245489
Axitinib	VEGFR inhibitor	RCC	III	Pembrolizumab	NCT02853331
		Advanced Melanoma	II	Nivolumab	NCT04493203
Azacitidine	DNA methyltransferase inhibitor	RCC	I/II	Pembrolizumab	NCT02959437
Bevacizumab	anti-VEGFR mAb	RCC	III	Atezolizumab	NCT02420821
		CRC	II		NCT02873195
		HCC	III		NCT03434379
		NSCLC	III		NCT02366143
		RCC	II		NCT04017455
		Hepatocellular carcinoma, Hepatocellular cancer	IV		NCT05185505
β-blocker	Adrenalin β-receptor inhibition	Melanoma	Ib/II	Pembrolizumab	NCT03384836
Carboplatin	DNA synthesis inhibitor	NSCLC	III	Atezolizumab	NCT02367781
		NSCLC	III	Atezolizumab	NCT02763579
		NSCLC	III	Pembrolizumab	NCT03434379
		NSCLC	III	Pembrolizumab	NCT02775435
		TNBC	II	Nivolumab	NCT03414684
Cabozantinib	Met, AXL, Ret, VEGFR2 inhibitor	Refractory cutaneous melanoma	II	Ipilimumab/Nivolumab	NCT05200143
		Carcinoid tumor	II	Nivolumab	NCT04197310
		Breast cancer	II	Nivolumab	NCT03316586
Capecitabine	DNA/RNA synthesis inhibitor	CRC		Atezolizumab	NCT02873195
Celecoxib	COX-2 inhibitor	Metastatic cancer	II	Nivolumab	NCT03864575
Cisplatin	DNA/RNA synthesis inhibitor	NSCLC		Pembrolizumab	NCT02578680
Epacadostat	IDO1 inhibitor	Gastric cancer/Esophageal cancer	II	Pembrolizumab	NCT03196232
		Head and Neck cancer	III		NCT03358472
		Lung cancer	II		NCT03322540
		Lung cancer	II		NCT03322566
		RCC	III		NCT03260894
Etoposide	Topoisomerase II inhibitor	NSCLC	III	Atezolizumab	NCT02763579
Foslinanib	Angiogenesis inhibitors	Advanced cancer, Hepatocellular carcinoma	II	Nivolumab	NCT05257590
	Apoptosis stimulants				
	Growth inhibitors				
HF10	Oncolytic virus	Melanoma	II	Ipilimumab	NCT02272855
				Ipilimumab	NCT03153085
				Nivolumab	NCT03259425
Lenvatinib	VEGFR2/3 inhibitor	Hepatocellular carcinoma	II/III	Nivolumab	NCT04044651
M7824	TGF-β/anti-PD-L1 bifunctional fusion protein	Solid tumor	I	N/A	NCT02517398
Metformin	pyruvate carboxylase inhibitor	Colorectal adenocarcinoma	II	Nivolumab	NCT03800602
		Colorectal carcinoma	II	Nivolumab	NCT03800602
		HNSCC	II	Pembrolizumab	NCT04414540
		NSCLC	II	Sintilimab	NCT03874000
NG - 641	Oncolytic virus	Metastatic cancer, Epithelial tumor	I	Nivolumab	NCT05043714
Regorafenib	VEGFR inhibitor	Hepatocellular carcinoma	I/II	Nivolumab	NCT04170556
Ramucirumab	anti-VEGFR2 mAb	Mesothelioma	II	Nivolumab	NCT03502746
Tivozanib (AV-951)	VEGFR inhibitor	RCC	I/II	Nivolumab	NCT03136627
		RCC	III	Nivolumab	NCT04987203
		Breast cancer, Ovarian cancer	I/II	Atezolizumab	NCT05000294
NAB-paclitaxel	Nanoparticle Albumin Bound	TNBC	III	Atezolizumab	NCT02425891
	Paclitaxel	NSCLC	III	Atezolizumab	NCT02367781
		NSCLC	III	Pembrolizumab	NCT02775435
		HNSCC	II	Nivolumab	NCT04831320
		HNSCC	II	Pembrolizumab	NCT04857164
NKTR-214	PEGylated IL-2	Melanoma, NSCLC	I/II	Nivolumab/Ipilimumab	NCT02983045
Pemetrexed	DNA replication inhibitor	NSCLC	III	Atezolizumab	NCT02367781
		NSCLC	III	Pembrolizumab	NCT02775435
Paclitaxel	Microtubule disassembly inhibition	NSCLC	III	Atezolizumab	NCT02366143
		SCLC	II	Pembrolizumab	NCT02551432
		Metastatic breast cancer	II	Pembrolizumab	NCT04251169
T-VEC	Oncolytic virus	Melanoma	I/II	Ipilimumab	NCT01740297
		Melanoma	III	Pembrolizumab	NCT02263508
		HNSCC	I	Pembrolizumab	NCT02626000

Tumor-associated macrophages are immune cells that regulate various factors in the tumor microenvironment and play an important role in connecting cancer with various immune cells in the tumor microenvironment (Pan et al., 2020; Zhou et al., 2020). Recently, these tumor-associated macrophages have also been recognized as biomarkers for the treatment of cancer, and anti-cancer drugs have been developed targeting them (Table 2). This new therapeutic approach is to achieve the effect of reprogramming immune cells to attack cancer cells by combination therapy with immune checkpoint inhibitors and a next-generation immuno-oncology drugs developed based on tumor-associated macrophages. The therapeutic strategy and development of drug targeting tumor-associated macrophages to date is limited to inhibiting the recruitment, polarization, and immune suppression of them. Thus, in order to develop a novel therapeutic strategy and development of drug that encompasses the whole tumor microenvironment, more in-depth research is essentially needed on detailed mechanisms in which macrophages regulate immunity.

**TABLE 2:** Tumor-associated Macrophage targeted therapy.

Drug	Mechanism	Indication	Clinical phase	Combination with ICI	Clinical trial no.
AMD3100	CXCR4 inhibitor	HNSCC	II	Pembrolizumab	NCT04058145
AMG820	anti-CSF-1R mAb	Advanced solid tumors	I/II	Pembrolizumab	NCT02713529
			I	N/A	NCT01444404
APX005M	CD40 agonist	Metastatic melanoma, NSCLC	I/II	Nivolumab	NCT03123783
		Advanced melanoma, RCC	I	Nivolumab, Ipilimumab	NCT04495257
		Metastatic pancreatic cancer, Advanced melanoma	I/II	Nivolumab	NCT03214250
		NSCLC, RCC	I	Nivolumab	NCT03502330
		Metastatic Melanoma	I	Nivolumab, Ipilimumab	NCT03597282
ARRY-382	CSF-1R Inhibitor	Melanoma, NSCLC, Solid tumors	I/II	Pembrolizumab	NCT02880371
BLZ945	CSF-1R Inhibitor	Solid tumors	I/II	PDR001	NCT02829723
BL-8040	CXCR4 inhibitor	Metastatic pancreatic adenocarcinoma	II	Pembrolizumab, Pembrolizumab	NCT02826486
		Metastatic recurrent or stage IV PDAC	I		NCT02907099
BMS-813160	CCR2/5 Inhibitor	Adanced RCC	II	Nivolumab, Ipilimumab, Relatlimab	NCT02996110
		Adanced RCC	II	Nivolumab	NCT02996110
		NSCLC, HCC	I/II	Nivolumab	NCT04123379
		Locally advanced PDAC	I/II	Nivolumab	NCT03767582
		Pancreatic Ductal Adenocarcinoma	I/II		NCT03496662
BNT411	TLR 7 agonist	NSCLC, Solid tumor	I/II	Atezolizumab	NCT04101357
Cabiralizumab	anti-CSF-1R mAb	Advanced HCC	II	Nivolumab	NCT04050462
		Resectable biopsiable BTC	II	Nivolumab	NCT03768531
		Solid tumors	I	Nivolumab	NCT02526017
		Peripheral T Cell Lymphoma	II	Nivolumab	NCT03927105
		Pancreatic Cancer	II	Nivolumab	NCT03599362
		Pancreatic Cancer	II	Nivolumab	NCT03697564
		TNBC	I/II	Nivolumab	NCT04331067
		Cancer	I	Nivolumab, Urelumab	NCT03431948
CC-90002 mAb	anti-CD47 mAb	Myeloid leukemia	I	N/A	NCT02641002
		Advanced solid hematologic cancer	I	Rituximab	NCT02367196
CNTO 888 (Carlumab)	anti-CCR2 mAb	Prostate cancer	II	N/A	NCT00992186
CC-90002 mAb	anti-CD47 mAb	Myeloid leukemia	I	N/A	NCT02641002
		Advanced solid hematologic cancer	I	Rituximab	NCT02367196
CNTO 888 (Carlumab)	anti-CCR2 mAb	Prostate cancer	II	N/A	NCT00992186
CP-870,893	CD40 agonist	Recurrent or stage IV melanoma	I	Tremelimumab	NCT01103635
		Advanced Solid tumors, Melanoma	I		NCT02225002
DCC-3014	CSF-1R Inhibitor	Metastatic solid tumors	I/II	N/A	NCT03069469
DSP-0509	TLR 7 agonist	Neoplasms	I/II	Pembrolizumab	NCT03416335
Duvelisib	PI3KƟ Inhibitor	HNSCC	I/II	Pembrolizumab	NCT04193293
Emactuzumab	anti-CSF-1R mAb	Advanced solid tumors	I	RO7009789	NCT02760797
		Solid tumors		Atezolizumab	NCT02323191
Evorpacept	CD47 inhibitor	HNC, HNSCC	II	Pembrolizumab	NCT04675294
Hu5F9-G4	anti-CD47 mAb	Ovariancancer, Solid tumors	I	Avelumab	NCT03558139
		Urothelial Carcinoma	I/II	Atezolizumab	NCT03869190
		Acute myeloid leukemia,	I	N/A	NCT02678338
		Myeloid leukemia	I	N/A	NCT02678338
IMC-CS4 (LY3022855 mAb)	Anti-CSF-1R mAb	Pancreatic cancer	I	Cyclophosphamide, GVAX, Pembrolizumab,	NCT03153410
IPI-549	PI3Kɤ Inhibitor	Avanced Solid Tumors	I	Nivolumab	NCT02637531
		Bladder Cancer, Urothelial Carcinoma	II	Nivolumab	NCT03980041
LHC165	TLR 7 agonist	Solid tumors	I	PDR001	NCT03301896
MCS110	Anti-CSF-1 mAb	Solid tumors	I/II	PDR001	NCT02807844
MLN1202	anti-CCR2 mAb	Bon metastasis	I/II	N/A plozalizumab	NCT01015560
		Melanoma	I		NCT02723006
NKTR-265	TLR 7/8 agonist	Solid tumors	I/II	Nivolumab, NKTR-214	NCT03435640
PD-0360324	anti-CSF-1 mAb	Solid tumors	I	Avelumab	NCT02554812
PF-04136309	CCR2 inhibitor	Pancreatic cancer	I/II	Nab-paclitaxel, Gemcitabine	NCT02732938
PLX7486 (Plexxikon)	CSF-1R Inhibitor	Advanced-stage solid tumors	I		
Pexidartinib(PLX3397)	CSF-1R Inhibitor	Advanced pancreatic cancer, CRC	I	Durvalumab	NCT02777710
		Acute myeloid leukemia	I/II	N/A	NCT01349049
		Advanced solid tumors,	I	N/A	NCT02734433
		GCT-TS, PVNS	III	N/A	NCT02371369
		Leukemia, Neurofibroma, Sarcoma	I/II	N/A	NCT02390752
		Melanoma	II	N/A	NCT02071940
Selicrelumab	CD40 agonist	Solid tumors	I	Atezolizumab	NCT02304393
SF1126	pan-PI3K Inhibitor	Advanced HCC	I	Nivolumab	NCT03059147
SRF231	anti-CD47 mAb	Advanced solid cancers hematologic cancers	I	N/A	NCT03512340
TTI-621	SIRP-ɑ Fc mAb	Solid tumors	I/II	anti-PD-1/L1 mAb	NCT02890368
		Hematologic Malignancies and Solid tumors	I	Nivolumab	NCT02663518
Zoledronate acid	Zoledronate acid	Kidney Cancer, Lung Metastases	I/II	Therapeutic autologous lymphocytes plus IL-2	NCT00588913
		Metastatic Kidney cancer	II	IL-2	NCT00582790

## Conclusion

Cancer can occur in otherwise healthy people because many tumors have specialized survival mechanisms to evade the immune response of the host. Furthermore, because tumor cells originally derive from the normal cells of the host, they still have many similar properties to normal cells, and their immunogenicity is lower than that of pathogenic microorganisms. Therefore, many naturally occurring tumors, not those caused by carcinogenic virus infections, only cause very weak immune responses. In tumors that proliferate or migrate quickly, the rate of proliferation and diffusion exceeds the rate of cancer cell removal by the immune system, so it cannot be eradicated by the immune response. Cancer immunologists are racing to increase the effectiveness of cancer treatment by reversing or neutralizing the immune evasion mechanism of these cancer cells.

Macrophages were originally known to directly kill non-self cancer cells, but recent studies have shown that tumor-associated macrophages promote the proliferation of cancer cells and help cancer cells that cause metastasis to pass through blood vessel walls easily. As the cancer mass grows, the immune response in the TME is severely inclined toward the suppression of the anti-cancer response by tumor-associated immune cells, especially tumor-associated macrophages, impeding the effectiveness of other treatments such as immunotherapy or chemotherapy. Explaining the role of macrophages in tumor biology is important because regulating their activity can open up new opportunities for therapeutic interventions. Similarly, it is also important to understand how macrophages in the TME are affected by current treatments aimed at cancer cells.
